# Differential Plasma Proteins Identified via iTRAQ-Based Analysis Serve as Diagnostic Markers of Pancreatic Ductal Adenocarcinoma

**DOI:** 10.1155/2023/5145152

**Published:** 2023-01-20

**Authors:** Xiubing Chen, Xiaomin Liao, Biaolin Zheng, Feng Wang, Feiran Chen, Zhejun Deng, Haixing Jiang, Shanyu Qin

**Affiliations:** Department of Gastroenterology, The First Affiliated Hospital of Guangxi Medical University, Nanning, Guangxi 530021, China

## Abstract

**Objective:**

We aimed to identify differentially expressed proteins in the plasma of patients with pancreatic cancer and control subjects, which could serve as potential tumor biomarkers.

**Methods:**

Differentially expressed proteins were determined via isostatic labeling and absolute quantification (iTRAQ). Potential protein biomarkers were identified via enzyme-linked immunosorbent assay (ELISA) in 40 patients and 40 control subjects, and those eventually selected were further validated in 40 pancreatic cancer and normal pancreatic tissues.

**Results:**

In total, 30 proteins displayed significant differences in expression among which 21 were downregulated and 9 were upregulated compared with the control group. ELISA revealed downregulation of peroxiredoxin-2 (PRDX2) and upregulation of alpha-1-antitrypsin (AAT), Ras-related protein Rab-2B (RAB2B), insulin-like growth factor-binding protein 2 (IGFBP2), Rho-related GTP-binding protein RhoC (RHOC), and prelamin-A/C (LMNA) proteins in 40 other samples of pancreatic cancer. Notably, only AAT, RAB2B, and IGFBP2 levels were consistent with expression patterns obtained with iTRAQ. Moreover, all three proteins displayed a marked increase in pancreatic cancer tissues. Data from ROC curve analysis indicated that the diagnostic ability of AAT, RAB2B, and IGFBP2 combined with carbohydrate antigen 19-9 (CA19-9) for pancreatic cancer was significantly greater than that of the single indexes (area under the curve (AUC): 90% vs. 75% (CA19-9), 76% (AAT), 71% (RAB2B), and 71% (IGFBP2), all *P* < 0.01).

**Conclusion:**

AAT, RAB2B, and IGFBP2 could serve as effective biomarkers to facilitate the early diagnosis of pancreatic cancer.

## 1. Introduction

Pancreatic ductal adenocarcinoma (PDAC), a highly malignant tumor with an annual incidence rate of 0.5% to 1% [1], has a 5-year survival rate of <10% [2]. The high mortality rate of PDAC is mainly attributable to the lack of clinical symptoms and detection markers in the early stages. Blood-borne markers are easy to obtain and detect; serve as excellent samples for a variety of diseases, including cancer; and are suitable for use in diagnosis and follow-up monitoring. Proteins circulating in the blood are essential nutrients for human life. Proteomics is a field of large-scale characterization of proteins that provides an effective technique for detection of biomarkers. Isobaric tag for relative and absolute quantification (iTRAQ) is an advanced quantitative proteomic approach that has been widely used in multiple fields for the diagnosis and treatment of numerous diseases since its conception in 2004 [3–5].

In this study, we utilized iTRAQ-based technology to detect differentially expressed proteins in plasma samples of PDAC patients, resulting in the identification of 30 proteins with potential significance. Through consultation of the relevant literature, six of the differentially expressed proteins were selected for further validation. Notably, levels of alpha-1-antitrypsin (AAT), Ras-related protein Rab-2B (RAB2B), and insulin-like growth factor-binding protein 2 (IGFBP2) were consistently increased in PDAC samples, indicative of their utility as potential biomarkers. Our collective findings showed enhanced diagnostic capacity of a combination of these novel protein markers with carbohydrate antigen 19-9 (CA19-9) for pancreatic cancer.

## 2. Materials and Methods

### 2.1. Patient Samples

Plasma samples from 16 patients with pancreatic cancer and 16 gender-matched age-matched noncancer controls at the First Affiliated Hospital of Guangxi Medical University (Nanning, China) were analyzed via mass spectrometry. Plasma samples of a further 40 patients with pancreatic cancer and 40 noncancer control subjects were obtained for ELISA. The clinical stages were classified according to the TNM classification of the Union for International Cancer Control [6]. All blood samples were collected from patients in the morning after an overnight fast. After incubation at room temperature for 1 hour, samples were centrifuged at 3000 g/min for 20 minutes. The supernatant was removed to a new EP tube and immediately stored at -80°C. The demographic characteristics of all participants are presented in Table 1.

### 2.2. Laboratory Assays

Serum CA19-9 levels of all participants were measured at admission. The results were provided by the laboratory department of the hospital.

### 2.3. Tissue Samples

Forty pancreatic cancer and normal pancreatic tissues were included for analysis. Written informed consent (No.: 2022(KY-E-037)) was obtained from all participants and approved by the ethics committee of the First Affiliated Hospital of Guangxi Medical University.

### 2.4. Sample Preparation and iTRAQ Labeling

Plasma samples from 16 pancreatic cancer patients and 16 control subjects were thawed at room temperature followed by centrifugation at 2000 g/min for 10 minutes at room temperature, and 100 *μ*L supernatant was collected from each tube. All 4 samples in the two groups were mixed into a single sample and subjected to mass spectrometry analysis. Plasma pools were depleted of most abundant proteins using a specific kit (Millipore, Inc., Massachusetts, USA; 122642) according to the manufacturer's instructions. Ultrafiltration tubes (3 kDa, Millipore) were used for desalination and concentration of low-abundance components. Protein in the supernatant was quantified with a BCA Protein Assay Kit (Thermo Scientific, Inc., Massachusetts, USA; 23225). An aliquot of protein (10 *μ*g) from each sample was mixed with 5x loading buffer, separated via 12% SDS-PAGE, and visualized using Coomassie blue staining. A filter-aided sample preparation (FASP) was used for removal of DTT and other low molecular weight components and digestion of proteins. Each peptide mixture was labeled using the iTRAQ Reagent 8-plex kit according to the manufacturer's instructions, lyophilized, and stored at -80°C.

### 2.5. Liquid Chromatography with Tandem Mass Spectrometry Analysis

Lyophilized peptide fractions were resuspended in 2% acetonitrile containing 0.1% formic acid. Aliquots of 4 *μ*L were loaded onto a ChromXP C18 (3 *μ*m, 150 Å) trap column and chromatographic separation performed on the Ekspert nanoLC 415 system (SCIEX, Concord, ON, Canada). The iTRAQ-labeled peptides were separated using analytical columns (ChromXP, Eksigent). High-resolution LC-MS/MS analysis was performed on a Q Exactive mass spectrometer (Thermo Scientific). The mass spectrometry scan was set to a full scan charge-to-mass ratio *m*/*z* range of 300-1600, and the 10 highest peaks were scanned via MS/MS. All MS/MS spectra were collected in the positive ion mode using data-dependent high-energy collisional fragmentation. Raw data were processed using Proteome Discoverer 2.4 (Thermo Scientific), and searches against the UniProt protein human database were performed using ProteinPilot software (version 5.0, SCIEX). The false discovery rate (FDR) for peptides was set at 1%.

### 2.6. Enzyme-Linked Immunosorbent Assay (ELISA)

AAT ELISA kit (MEIMIAN, Inc., Yancheng, China; http://www.mmbio.cn, MM-50791H1), RAB2B ELISA kit (MEIMIAN, Inc., MM-60064H1), IGFBP2 ELISA kit (MEIMIAN, Inc., MM-60020H1), PRDX2 ELISA kit (MEIMIAN, Inc., MM-51835H1), RHOC ELISA kit (MEIMIAN, Inc., MM-60061H1), and LMNA ELISA kit (MEIMIAN, Inc., MM-60022H1) were employed to determine the levels of AAT, RAB2B, IGFBP2, PRDX2, RHOC, and LMNA in plasma. The experimental procedures rigorously followed the manufacturer's instructions.

### 2.7. Immunohistochemical Analysis

Sections of formalin-fixed and paraffin-embedded tissues were analyzed via immunohistochemistry (IHC). In brief, slices were incubated with 3% hydrogen peroxide solution to block endogenous peroxidase activity; treated with boiling citrate buffer, pH 6.0 (Wuxi Aorui Dongyuan Biotechnology Co., Ltd., Wuxi, China; ZLI-9065), for 6 minutes for antigen repair; and blocked with 10% goat serum (Solarbio Co., Dalian, China, SL038) for 20 minutes. Next, sections were incubated with anti-AAT (Proteintech Group Inc., Wuhan, China; 66135-1-lg, 1 : 300), anti-RAB2B (Proteintech Group Inc., 11756-1-ap, 1 : 250), and anti-IGFBP2 (Beijing BIOSS Biotechnology Co., Ltd., Beijing, China; bs-1108r, 1 : 100) antibodies at 37°C for 1 hour, followed by enhanced anti-rabbit biotin IgG (Beijing Zhongshan Jinqiao Biotechnology Co., Ltd., Beijing, China; PV-9000) for 20 minutes, and finally DAB solution (Beijing Zhongshan Jinqiao Biotechnology Co., Ltd.; ZLI-9017) for color development for AAT (90 seconds), RAB2B (120 seconds), and IGFBP2 (120 seconds). Stained sections were identified and scored by two high-level pathologists as follows: staining area score < 26% (1 point), 26-50% (2 points), 51-75% (3 points), or 76-100% (4 points) and staining intensity score of light yellow (1 point), brownish yellow (2 points), or brown (3 points). The final score was calculated as the staining area score multiplied by staining intensity score [7].

### 2.8. Statistical Analysis

The Kolmogorov-Smirnov test was used to determine the normality of continuous data. Normally, distributed variables were expressed as mean values ± standard deviation (SD) and compared with the Student *t*-test. Categorical variables were expressed in absolute numbers and/or percentage frequencies and compared using chi-square or Fisher's exact test, as required. Logistic regression and positive likelihood ratio test were used to conduct multivariate analysis. The receiver operating curve (ROC) and area under the curve (AUC) were calculated to evaluate the ability of individual or multiple markers to diagnose PDAC. All statistical analyses were performed using SPSS software (version 25.0; SPSS Inc., Chicago, Illinois, USA). A two-tailed *P* value < 0.05 was considered significant.

## 3. Results

### 3.1. Quantitative Proteomic Analysis of Plasma via iTRAQ

**Figure 1:**
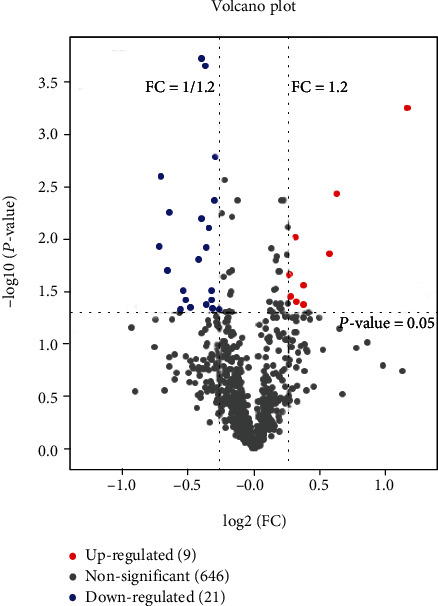
Differentially expressed plasma proteins in PDAC patients detected via iTRAQ.

iTRAQ technology was employed to analyze the differentially expressed plasma proteins between pancreatic cancer and control groups. In total, 676 proteins were detected in blood samples (Figure 1).

Using a cutoff threshold of >1.2 or <0.8 and 95% confidence level of 1% FDR [8], we identified 30 proteins showing significantly different expression between PDAC patients and noncancer controls. Among these, 21 proteins were downregulated and 9 were upregulated (Table 2).

### 3.2. Candidate Biomarker Verification

**Figure 2:**
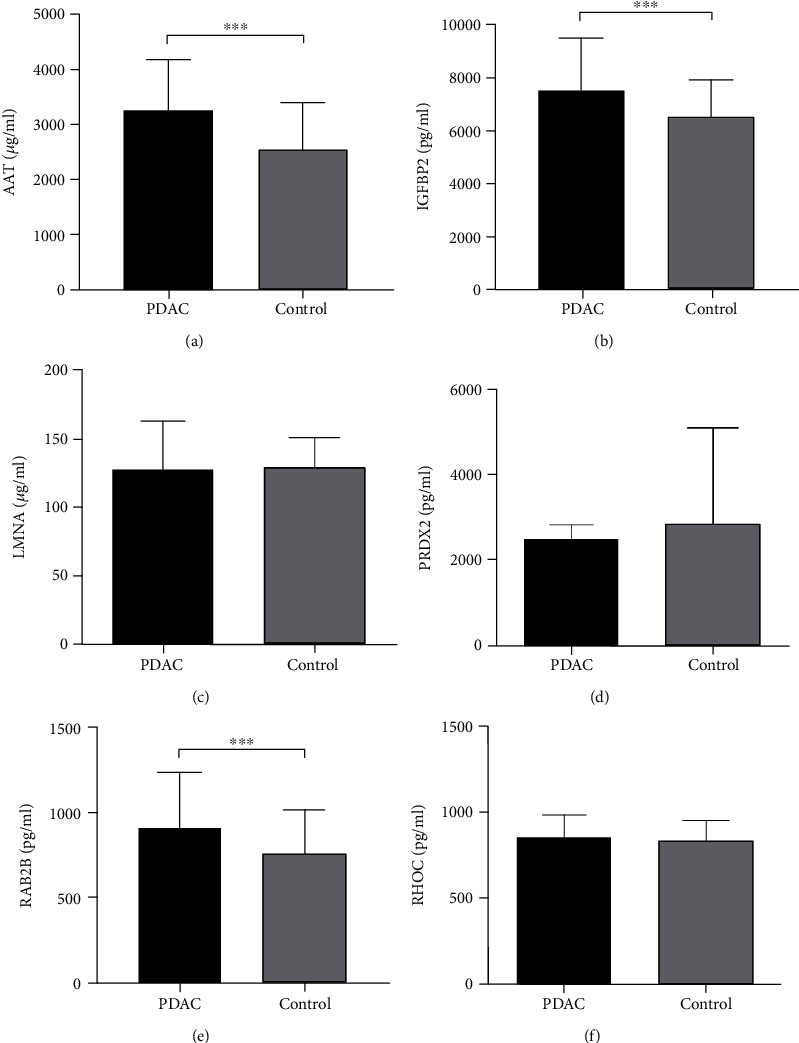
Validation of six differentially expressed proteins in PDAC plasma with ELISA. Plasma levels of (a) AAT, (b) IGFBP2, (c) LMNA, (d) PRDX2, (e) RAB2B, and (f) RHOC in PDAC patients and normal controls. ^∗∗∗^*P* < 0.001 vs. control.

ELISA was employed to validate differentially expressed proteins in plasma of PDAC samples. To verify iTRAQ results, we further examined the expression of six candidate proteins (AAT, IGFBP2, RAB2B, PRDX2, RHOC, and LMNA) in plasma samples of 40 additional PDAC patients and 40 control subjects. Notably, AAT, IGFBP2, and RAB2B expressions in pancreatic cancer patients were markedly higher relative to that in the control group while no significant differences were evident in PRDX2, RHOC, and LMNA levels in our ELISA experiments. Our results indicate that PRDX2, RHOC, and LMNA are not useful biomarkers for detection of PDAC (Figure 2(a)–2(f)).

**Figure 3:**
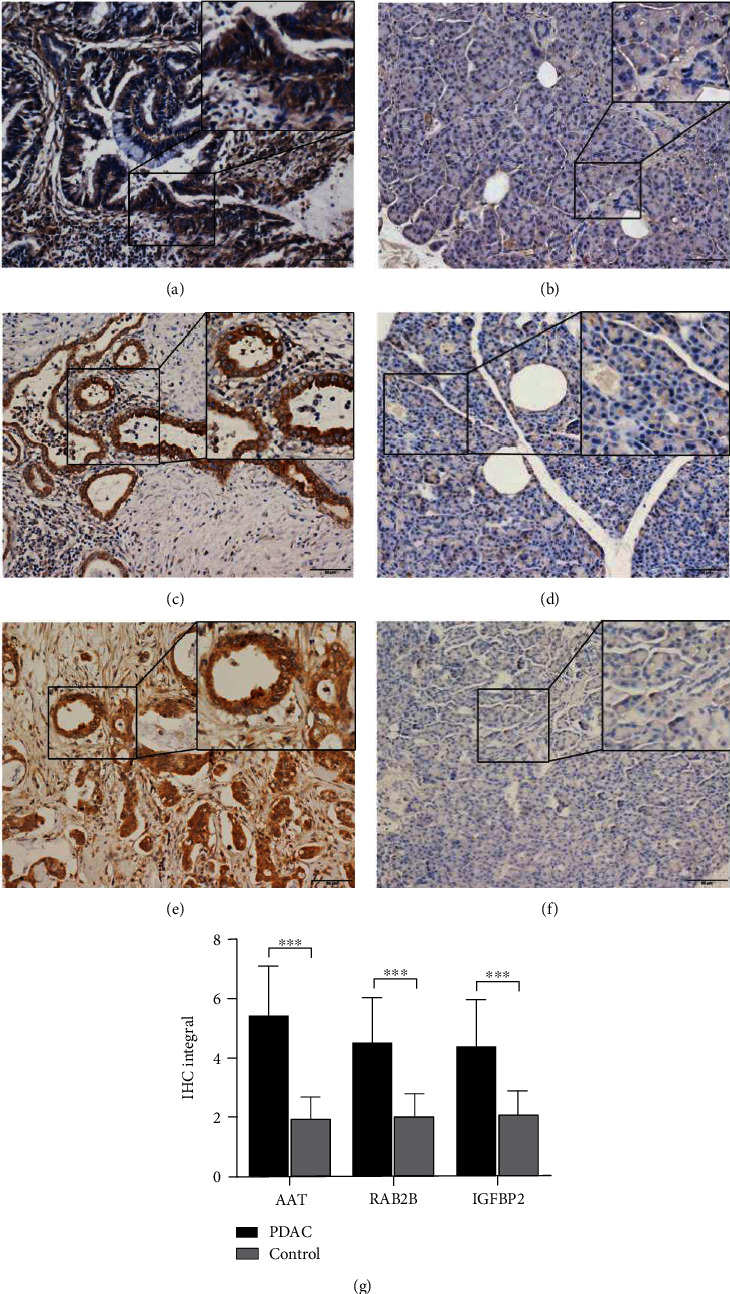
IHC detection of differential protein expression in PDAC tissue sections. (a) AAT levels in PDAC patients. (b) AAT levels in normal controls. (c) RAB2B levels in PDAC patients. (d) RAB2B levels in normal controls. (e) IGFBP2 levels in PDAC patients. (f) IGFBP2 levels in normal controls. (g) IHC integral analysis of AAT, RAB2B, and IGFBP2 in PDAC patients and normal controls.

Consistently, among 40 pairs of pancreatic cancer and normal pancreas tissues, IHC scores of AAT, RAB2B, and IGFBP2 were significantly higher in pancreatic cancer than the control group (Figures 3(a)–3(g)).

Next, receiver operating characteristic (ROC) analysis was performed to evaluate the diagnostic performance of AAT, RAB2B, and IGFBP2 as biomarkers and calculate the threshold values (Table 3). Based on ROC curves, AUC values of 0.755 (95% CI, 0.650-0.860), 0.712 (95% CI, 0.597-0.827), and 0.714 (95% CI, 0.601-0.827) were obtained for AAT, RAB2B, and IGFBP2, respectively. The AUC values, sensitivity, and specificity of AAT were similar to those of CA19-9, and those of RAB2B and IGFBP2 were lower, but specificities were greater than those of CA19-9.

Thresholds for AAT, RAB2B, and IGFBP2 were determined as the points with minimum distance from 100% sensitivity and 100% specificity in the ROC curve for PDAC (*n* = 40) and noncancer controls (*n* = 40). The threshold for CA19-9 is the standard value for clinical diagnosis. AUC is the area under the ROC curve, and the range of 95% CI is shown. The odds ratio was calculated as %sensitivity × %specificity/(100 − %sensitivity) × (100 − %specificity).

To assess compensatory ability for CA19-9, the values of each marker in CA19-9-negative patients (<37 U/mL) were evaluated, as shown in Table 4. Among 12 CA19-9-negative patients, 7, 6, and 5 were AAT-, RAB2B-, and IGFBP2-positive, respectively, suggestive of compensatory abilities of the three markers for CA19-9.

**Figure 4:**
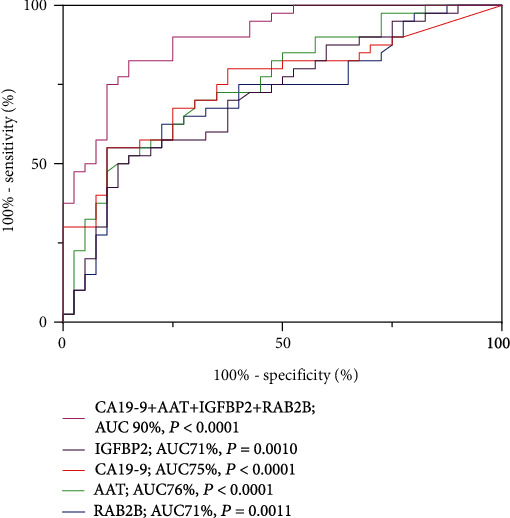
Receiver operating characteristic (ROC) curves of the four indicators.

To evaluate the diagnostic ability of plasma AAT, RAB2B, and IGFBP2 in combination with CA19-9 for pancreatic cancer, ROC curves of the 4 indicators were calculated for determining their individual and combined values in diagnosis. Our results showed that the diagnostic ability of the combined index comprising all four parameters was significantly greater than that of the single indexes (Figure 4).

### 3.3. The Exploration of AAT, RAB2B, and IGFBP2 as Plasma Biomarkers in the Diagnosis of Early-Stage PDAC

We identified 15 stage I or II PDAC patients from 40 PDAC patients with ELISA testing (Table 5) and formed a dataset with 40 noncancer control subjects with ELISA testing, in order to verify whether AAT, RAB2B, and IGFBP2 can be used as plasma markers in the diagnosis of early-stage PDAC.

**Figure 5:**
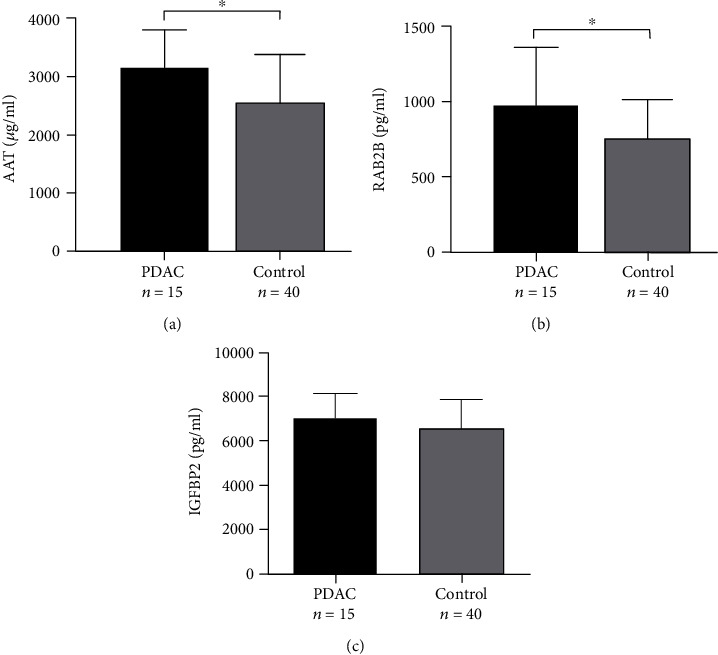
Validation of three differentially expressed proteins in early-stage PDAC plasma with ELISA. Plasma levels of (a) AAT, (b) RAB2B, and (c) IGFBP2 in early-stage PDAC patients and normal controls. ^∗^*P* < 0.05 vs. control.

As shown in Figure 5, the quantitative values of AAT and RAB of early-stage PDAC patients were significantly greater than those of the controls (Figures 5(a) and 5(b)). Unfortunately, there were no significant differences between the quantitative values of IGFBP2 and controls (Figure 5(c)).

**Figure 6:**
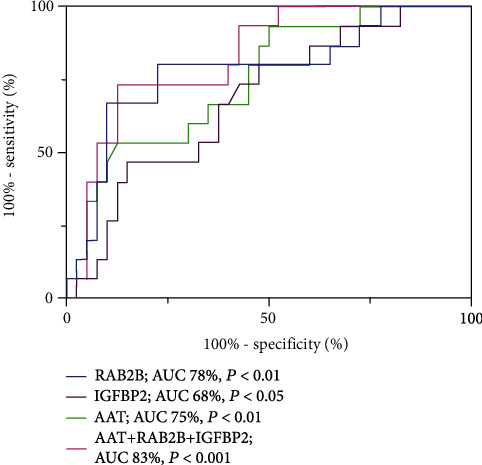
Receiver operating characteristic (ROC) curves showed the diagnostic performance of AAT, RAB2B, and IGFBP2 as early-stage PDAC biomarkers individually or jointly.

Then, we conducted ROC analysis to evaluate the diagnostic performance of AAT, RAB2B, and IGFBP2 as early-stage PDAC biomarkers individually or jointly. According to ROC curves, the AUC values of AAT, RAB2B, and IGFBP2 were 0.751 (95% CI, 0.612-0.890), 0.780 (95% CI, 0.632-0.928), and 0.683 (95% CI, 0.530-0.835), respectively. The AUC value of the combination of AAT, RAB2B, and IGFBP2 was 0.830 (95% CI, 0.716-0.945) (Figure 6).

## 4. Discussion

The plasma proteome profiles of patients with pancreatic cancer were compared with those of noncancer controls in an attempt to develop a noninvasive diagnostic test for pancreatic cancer. Using iTRAQ-based two-dimensional LC-MS/MS analysis, we initially identified 30 differentially expressed proteins in sera of patients, which were associated with protease inhibition, regulation of protein secretion, antioxidation, tumor control, and lipid metabolism. Among these proteins, six (AAT, IGFBP2, RAB2B, PRDX2, RHOC, and LMNA) with functions closely related to pancreatic disease were selected for further validation. Notably, ELISA results were consistent with iTRAQ data for AAT, IGFBP2, and RAB2B. Subsequent evaluation of the expression patterns of these three proteins in 40 pairs of pancreatic cancer and corresponding normal pancreatic tissues confirmed significantly higher AAT, RAB2B, and IGFBP2 levels in pancreatic cancer tissues relative to normal pancreatic tissue samples.

AAT encoded by *SERPINA1* is a serine protease inhibitor mainly synthesized by the liver. This highly expressed glycoprotein is released into the bloodstream [9] and acts as an inhibitor of neutrophil elastase, trypsin, chymotrypsin, thrombin, plasmin, and cathepsin. Lack of AAT mainly triggers chronic obstructive pulmonary disease [10]. Neutrophils are known to play a key role in acute inflammatory disease development [9, 11]. The neutrophil-derived granule protein elastase and its neutrophil extracellular traps (NETs) cause serious tissue damage [12]. Accordingly, AAT has been characterized as an acute response protein in the past [13]. However, long-term imbalance of protease and AAT was subsequently shown to induce chronic tissue and cell damage [14–16]. Recent studies indicate that AAT can be effectively used as a diagnostic and prognostic marker for various tumor types, including small-cell lung cancer, breast cancer, gastric cancer, and colon cancer [17–20]. AAT acts on FN1 through Snail in colon and gastric cancer, resulting in epithelial-mesenchymal transformation, in turn, promoting cancer progression and metastasis [18, 20]. In view of the elevated levels of AAT in both chronic pancreatitis and PDAC samples, we speculate that AAT also induces progression of chronic pancreatitis to PDAC through this mechanism [15, 21]. The collective findings indicate that AAT not only is an acute phase reactive protein but also plays an important role in the acute pancreatitis-chronic pancreatitis-pancreatic cancer axis. As mentioned earlier, AAT is elevated in a number of diseases, including malignant tumors. Therefore, specific detection of PDAC is unlikely to augment diagnostic capability, and combination with other indicators is required to improve accuracy and specificity.

The Rab family of proteins, alternatively known as small GTP-binding proteins, is a member of the Ras-like small GTPase superfamily. Rab proteins serve as key regulators of intracellular transport and exert carcinogenic effects [22–24]. However, due to the large number of Rab isomers in mammals, the effector molecules and their binding specificities remain to be clarified [22, 25]. RAB2B, a member of the Rab family containing a conserved GTP-binding domain and variable N-terminal and C-terminal domains [22, 26], has been characterized as a protooncogene dysregulated in a variety of tumors and critical for tumor development [23, 27], showing a gradual decrease in expression with improvement of disease [28]. RAB2B is overexpressed in cervical cancer and interacts with IGF2BP3 to promote cell growth and proliferation [29]. Recent findings suggest that abnormal expression of mir-448 induces downregulation of RAB2B, significant reduction of pancreatic cancer cell proliferation, and promotion of cancer cell apoptosis [25]. However, the expression patterns of RAB2B in blood and pathological tissues of pancreatic cancer patients have not been reported to date. Our results further demonstrate that RAB2B is closely associated with pancreatic cancer. Further research on the specific role of RAB2B in pancreatic cancer is warranted to improve diagnostic accuracy and treatment strategies.

Insulin-like growth factor-binding protein 2 (IGFBP2) is composed of 328 residues with a molecular weight of 36 kDa. IGFBP2 has been identified as one of the six proteins in this family and is the second most abundant circulating IGFBP, but its physiological role remains unclear at present [30]. Previous studies suggest that IGFBP2 plays a role in diabetes and obesity mainly via regulation of IGFBP1 [31, 32]. However, subsequent findings indicate that increased IGFBP2 levels are associated with not only endocrine disorders but also development of multiple cancer types [33, 34]. IGFBP2 has been identified as a potential biomarker of pancreatic cancer [35]. Expression of IGFBP2 in pancreatic fluid, tissue, and plasma of PDAC patients is increased [36, 37] in correlation with tumor stage. IGFBP2 is reported to play a carcinogenic role through multiple signal pathways or specific factors, including p53 [38], PTEN and PI3K/Akt [39], Hedgehog (Hh) [40], and VEGF [41]. Analysis of these mechanisms has further established IGFBP2 as an important reference factor for the diagnosis and treatment of pancreatic cancer [42]. However, IGFBP2 alone is insufficient for effective prediction of pancreatic cancer and needs to be combined with other indicators to improve accuracy and specificity of diagnosis [37].

Serum carbohydrate antigen 19-9 (CA19-9) is the only recognized biomarker for the diagnosis of pancreatic cancer [43], with a sensitivity of 79-81% and specificity of 82-90% [44]. However, CA19-9 is also increased in benign pancreatic and biliary diseases, such as obstructive jaundice, pancreatitis, cholangitis, and cancer of the stomach, colon, ovary, uterus, liver, and other organs [45, 46]. In addition, 8-10% Caucasians with Lewis A-B genotype do not express CA19-9, indicative of limitations as a biomarker of pancreatic cancer [21].

ROC analysis revealed that the AUC values of CA19-9, AAT, RAB2B, and IGFBP2 were not significantly different, ranging from 71 to 76% (Table 3). However, among the 12 CA19-9-negative patients, 7 were AAT-positive, 6 were RAB2B-positive, and 5 were IGFBP2-positive (Table 4). Furthermore, collective application of AAT, RAB2B, IGFBP2, and CA19-9 in the diagnosis of pancreatic cancer was significantly more effective than each single index alone. These results suggest that AAT, RAB2B, and IGFBP2 have a compensatory ability for CA19-9, and combined usage of the four indicators should improve the accuracy of clinical PDAC diagnosis. Since the liver, bile duct, and pancreas have a common embryologic origin [6], AAT and IGFBP2 have the same limitations as CA19-9 in differentiating between liver cancer and cholangiocarcinoma. It is exciting that the combination of AAT, RAB2B, and IGFBP2 has a good effect on the diagnosis of early-stage PDAC (Figure 6). The plasma levels of AAT and RAB2B in early-stage PDAC patients were significantly higher than those in the noncancer control subjects (Figure 5). However, the expression level of IGFBP2 was not significantly increased as described by Yoneyama et al. [6], which might be the result of a different sample size or different regional population characteristics. Our findings disclosed elevated blood levels of three indicators (AAT, RAB2B, and IGFBP2), which could be used as a signature biomarker for auxiliary diagnosis via liquid biopsy and as a noninvasive monitoring indicator for prognostic follow-up. In addition, elevated expression of these three indicators was consistently observed in PDAC tissue samples, which could have utility in endosonography with fine-needle aspiration biopsy (EUS-FNA) and routine histopathological immunohistochemical detection, providing a multiway detection system for the diagnosis of PDAC.

## 5. Conclusion

In conclusion, AAT, RAB2B, and IGFBP2 were identified as potential biomarkers of pancreatic cancer through plasma proteomic iTRAQ analysis and further validated using ELISA and immunohistochemistry. To our knowledge, RAB2B is the first reported biomarker that may effectively facilitate the diagnosis of pancreatic cancer. Furthermore, the diagnostic capability of combined AAT, RAB2B, IGFBP2, and CA19-9 for pancreatic cancer was significantly improved. Data from this study provide valuable insights that could be utilized to develop a novel clinical strategy for the early detection of pancreatic cancer.

## Figures and Tables

**Table 1:** Clinical characteristics of patients included for iTRAQ and ELISA analyses.

Characteristics	iTRAQ		ELISA		*P* value
	Controls (*n* = 16)	PDAC (*n* = 16)	Controls (*n* = 40)	PDAC (*n* = 40)	
Sex M/F	9/7	10/6^∗^	18/22	19/21^#^	^∗^ *P* = 0.719 ^#^*P* = 0.823
Age (yrs), mean ($\bar{x}\pm s$)	55.75 ± 6.904	61.06 ± 9.037^∗^	58.65 ± 11.720	59.63 ± 9.339^#^	^∗^ *P* = 0.072 ^#^*P* = 0.682
BMI (kg/m^2^), mean ($\bar{x}\pm s$)	22.09 ± 2.594	20.57 ± 2.881^∗^	21.05 ± 3.528	20.34 ± 2.927^#^	^∗^ *P* = 0.128 ^#^*P* = 0.329

BMI: body mass index; ^∗^*P* for iTRAQ and ^#^*P* for ELISA.

**Table 2:** List of the 30 differentially expressed proteins in PDAC.

No.	Accession	Description	Coverage (%)	Unique peptides	*P* value	Fold change (cancer/control)
1	P02679	Fibrinogen gamma chain (FGG)	56	30	0.03775	0.802208
2	P06727	Apolipoprotein A-IV (APOA4)	76	33	0.006425	0.759622
3	P68871	Hemoglobin subunit beta (HBB)	95	8	0.011649	0.605902
4	P01859	Immunoglobulin heavy constant gamma-2 (IGHG2)	53	7	0.007808	0.79011
5	P69905	Hemoglobin subunit alpha (HBA1)	92	11	0.019823	0.634655
6	P0DOX5	Immunoglobulin gamma-1 heavy chain (fragment)	41	7	0.000223	0.772435
7	P02042	Hemoglobin subunit delta (HBD)	76	5	0.005568	0.640484
8	Q16610	Extracellular matrix protein 1 (ECM1)	22	11	0.001641	0.81717
9	P32119	Peroxiredoxin-2 (PRDX2)	23	4	0.015671	0.746562
10	P69891	Hemoglobin subunit gamma-1 (HBG1)	44	5	0.03102	0.689903
11	Q13885	Tubulin beta-2A chain (TUBB2A)	12	1	0.011906	0.778568
12	P00918	Carbonic anhydrase 2 (CA2)	20	5	0.00019	0.759015
13	P18428	Lipopolysaccharide-binding protein (LBP)	7	3	0.038152	0.700106
14	Q02985	Complement factor H-related protein 3 (CFHR3)	6	1	0.045369	0.806503
15	Q15365	Poly(rC)-binding protein 1 OS=Homo sapiens (PCBP1)	13	2	0.046226	0.677007
16	P07195	L-Lactate dehydrogenase B chain (LDHB)	7	2	0.030936	0.79955
17	P24821	Tenascin (TNC)	2	3	0.046769	0.832112
18	P78371	T-Complex protein 1 subunit beta (CCT2)	4	1	0.045181	0.716738
19	P21796	Voltage-dependent anion-selective channel protein 1 (VDAC1)	7	1	0.004223	0.812415
20	P52272	Heterogeneous nuclear ribonucleoprotein M (HNRNPM)	2	1	0.042055	0.778395
21	Q5T619	Zinc finger protein 648 (ZNF648)	2	1	0.002492	0.612377
22	P01009	Alpha-1-antitrypsin (SERPINA1)	68	35	0.035057	1.212389
23	P02748	Complement component C9 (C9)	32	17	0.021767	1.20568
24	P02545	Prelamin-A/C (LMNA)	8	3	0.003694	1.544529
25	P18065	Insulin-like growth factor-binding protein 2 (IGFBP2)	14	4	0.039877	1.247822
26	Q8WUD1	Ras-related protein Rab-2B OS=Homo sapiens (RAB2B)	7	1	0.013881	1.488336
27	P30086	Phosphatidylethanolamine-binding protein 1 (PEBP1)	6	1	0.009546	1.239642
28	P08134	Rho-related GTP-binding protein RhoC (RHOC)	5	1	0.027416	1.295181
29	P84243	Histone H3.3 (H3-3A)	5	1	0.000554	2.23796
30	Q5T011	KICSTOR complex protein SZT2 (SZT2)	0	1	0.042225	1.297243

**Table 3:** Receiver operating characteristics (ROC) of markers for PDAC.

Name	Sensitivity (%)	Specificity (%)	Odds ratio	AUC (95% CI)	Threshold
CA19-9	70.0%	70.0%	2.33	0.750 (0.641-0.859)	>37 U/mL
AAT	70.0%	70.0%	2.33	0.755 (0.650-0.860)	>2933 *μ*g/mL
RAB2B	55.0%	90.0%	5.50	0.712 (0.597-0.827)	>836.9 pg/mL
IGFBP2	52.5%	85.0%	3.50	0.714 (0.601-0.827)	>7083 pg/mL

**Table 4:** Levels of markers in plasma of CA19-9-negative PDAC patients.

Patient number	CA19-9 (U/mL)	AAT (*μ*g/mL)	RAB2B (pg/mL)	IGFBP2 (pg/mL)
1	2.13	**2959.17** ^∗^	**920.63** ^∗^	5755.00
2	2.55	2642.50	637.29	6680.00
3	2.30	**3009.17** ^∗^	745.63	**7538.33** ^∗^
4	2.90	**3588.33** ^∗^	788.33	6721.67
5	17.90	**3130.00** ^∗^	**865.42** ^∗^	6255.00
6	13.03	**3392.50** ^∗^	**900.83** ^∗^	**7821.67** ^∗^
7	4.49	**4559.17** ^∗^	617.50	**9646.67** ^∗^
8	14.67	2656.43	**2198.27** ^∗^	6502.50
9	9.61	**3117.14** ^∗^	756.92	**9352.50** ^∗^
10	19.44	2906.43	**941.54** ^∗^	**7302.50** ^∗^
11	3.73	1860.00	781.92	5590.00
12	2.00	1667.14	**1040.58** ^∗^	6152.50

Marker levels in plasma of CA19-9-negative PDAC patients are shown. The values in bold with asterisks are above the thresholds for AAT, RAB2B, and IGFBP2 presented in Table 3.

**Table 5:** Levels of markers in plasma of early-stage PDAC patients.

Patient number	Stage	AAT (*μ*g/mL)	RAB2B (pg/mL)	IGFBP2 (pg/mL)
1	I	3259.17	1128.96	7630.00
2	II	4224.29	613.65	6702.50
3	II	2692.14	1271.35	7777.50
4	II	2656.43	2198.27	6502.50
5	II	4113.57	905.96	7465.00
6	II	3606.43	941.54	7302.50
7	II	2906.43	781.92	5590.00
8	II	1860.00	1029.04	7102.50
9	II	2935.00	1040.58	6152.50
10	II	3438.57	852.12	10390.00
11	II	2531.43	684.81	6627.50
12	II	3346.67	1019.58	6755.00
13	II	3642.50	645.63	7146.67
14	II	3588.33	788.33	6721.67
15	II	2717.50	865.42	5946.67

## Data Availability

The data used to support the findings of this study are available from the corresponding authors upon request.

## References

[1] Park W., Chawla A., O'Reilly E. M. (2021). Pancreatic cancer: a review. *JAMA*.

[2] Siegel R. L., Miller K. D., Jemal A. (2020). Cancer statistics, 2020. *CA: a Cancer Journal for Clinicians*.

[3] Zhou F., Chen X., Chen G., Yan J., Xiao Y. (2019). Identification of SAA and ACTB as potential biomarker of patients with severe HFMD using iTRAQ quantitative proteomics. *Clinical Biochemistry*.

[4] Zhang Y., Ying X., Zhao Q. (2020). Identification of protein expression changes in hepatocellular carcinoma through iTRAQ. *Disease Markers*.

[5] Wang D., Chen J., Han J. (2022). iTRAQ and two-dimensional-LC-MS/MS reveal NAA10 is a potential biomarker in esophageal squamous cell carcinoma. *Proteomics. Clinical Applications*.

[6] Yoneyama T., Ohtsuki S., Honda K. (2016). Identification of IGFBP2 and IGFBP3 as compensatory biomarkers for CA19-9 in early-stage pancreatic cancer using a combination of antibody-based and LC-MS/MS-based proteomics. *PLoS One*.

[7] Creytens D. (2019). NKX2.2 immunohistochemistry in the distinction of Ewing sarcoma from cytomorphologic mimics: diagnostic utility and pitfalls—comment on Russell-Goldman et al. *Cancer Cytopathology*.

[8] Unwin R. D., Griffiths J. R., Whetton A. D. (2010). Simultaneous analysis of relative protein expression levels across multiple samples using iTRAQ isobaric tags with 2D nano LC-MS/MS. *Nature Protocols*.

[9] Pastor C. M., Vonlaufen A., Georgi F., Hadengue A., Morel P., Frossard J. L. (2006). Neutrophil depletion--but not prevention of Kupffer cell activation--decreases the severity of cerulein-induced acute pancreatitis. *World Journal of Gastroenterology*.

[10] Tan L., Dickens J. A., Demeo D. L. (2014). Circulating polymers in *α*_1_-antitrypsin deficiency. *The European Respiratory Journal*.

[11] Abdulla A., Awla D., Thorlacius H., Regnér S. (2011). Role of neutrophils in the activation of trypsinogen in severe acute pancreatitis. *Journal of Leukocyte Biology*.

[12] Merza M., Hartman H., Rahman M. (2015). Neutrophil extracellular traps induce trypsin activation, inflammation, and tissue damage in mice with severe acute pancreatitis. *Gastroenterology*.

[13] Rompianesi G., Hann A., Komolafe O., Pereira S. P., Davidson B. R., Gurusamy K. S. (2018). Serum amylase and lipase and urinary trypsinogen and amylase for diagnosis of acute pancreatitis. *Cochrane Database of Systematic Reviews*.

[14] Kavutharapu S., Nagalla B., Abbagani V. (2012). Role of proteases and antiprotease in the etiology of chronic pancreatitis. *Saudi Journal of Gastroenterology*.

[15] Mihas A. A., Hirschowitz B. I. (1976). Alpha-antitrypsin and chronic pancreatitis. *Lancet*.

[16] Kilty S. J., Bosse Y., Cormier C., Endam L. M. (2010). Polymorphisms in the SERPINA1 (alpha-1-antitrypsin) gene are associated with severe chronic rhinosinusitis unresponsive to medical therapy. *American Journal of Rhinology & Allergy*.

[17] Chan H. J., Li H., Liu Z., Yuan Y. C., Mortimer J., Chen S. (2015). SERPINA1 is a direct estrogen receptor target gene and a predictor of survival in breast cancer patients. *Oncotarget*.

[18] Kwon C. H., Park H. J., Choi J. H. (2015). Snail and serpinA1 promote tumor progression and predict prognosis in colorectal cancer. *Oncotarget*.

[19] Najafi Z., Mohamadnia A., Ahmadi R. (2020). Proteomic and genomic biomarkers for non-small cell lung cancer: peroxiredoxin, haptoglobin, and alpha-1 antitrypsin. *Cancer Medicine*.

[20] Yang J., Xiong X., Wang X., Guo B., He K., Huang C. (2015). Identification of peptide regions of SERPINA1 and ENOSF1 and their protein expression as potential serum biomarkers for gastric cancer. *Tumour Biology*.

[21] Wu C. C., Lu Y. T., Yeh T. S., Chan Y. H., Dash S., Yu J. S. (2021). Identification of fucosylated SERPINA1 as a novel plasma marker for pancreatic cancer using lectin affinity capture coupled with iTRAQ-based quantitative glycoproteomics. *International Journal of Molecular Sciences*.

[22] Fukuda M., Kanno E., Ishibashi K., Itoh T. (2008). Large scale screening for novel Rab effectors reveals unexpected broad Rab binding specificity. *Molecular & Cellular Proteomics*.

[23] Bryant K. L., Der C. J. (2017). Mutant RAS calms stressed-out cancer cells. *Developmental Cell*.

[24] Dong Q., Fu L., Zhao Y. (2017). Rab11a promotes proliferation and invasion through regulation of YAP in non-small cell lung cancer. *Oncotarget*.

[25] Jin J., Wu Y., Zhou D., Sun Q., Wang W. (2018). miR‑448 targets Rab2B and is pivotal in the suppression of pancreatic cancer. *Oncology Reports*.

[26] Tisdale E. J., Bourne J. R., Khosravi-Far R., Der C. J. (1992). GTP-binding mutants of rab1 and rab2 are potent inhibitors of vesicular transport from the endoplasmic reticulum to the Golgi complex. *The Journal of Cell Biology*.

[27] Qin X., Wang J., Wang X., Liu F., Jiang B., Zhang Y. (2017). Targeting Rabs as a novel therapeutic strategy for cancer therapy. *Drug Discovery Today*.

[28] Culine S., Honore N., Closson V. (1994). A small GTP-binding protein is frequently overexpressed in peripheral blood mononuclear cells from patients with solid tumours. *European Journal of Cancer*.

[29] Hu Y., Li Y., Huang Y. (2020). METTL3 regulates the malignancy of cervical cancer via post-transcriptional regulation of RAB2B. *European Journal of Pharmacology*.

[30] Cohen P., Ocrant I., Fielder P. J. (1992). Insulin-like growth factors (igfs): Implications for aging. *Psychoneuroendocrinology*.

[31] Kwon J., Stephan S., Mukhopadhyay A. (2009). Insulin receptor substrate-2 mediated insulin-like growth factor-I receptor overexpression in pancreatic adenocarcinoma through protein kinase C*δ*. *Cancer Research*.

[32] El-Mesallamy H. O., Hamdy N. M., Zaghloul A. S. (2013). Clinical value of circulating lipocalins and insulin-like growth factor axis in pancreatic cancer diagnosis. *Pancreas*.

[33] Barghash A., Helms V., Kessler S. M. (2015). Overexpression of IGF2 mRNA-binding protein 2 (IMP2/p62) as a feature of basal-like breast cancer correlates with short survival. *Scandinavian Journal of Immunology*.

[34] Dai N., Ji F., Wright J., Minichiello L., Sadreyev R., Avruch J. (2017). IGF2 mRNA binding protein-2 is a tumor promoter that drives cancer proliferation through its client mRNAs IGF2 and HMGA1. *eLife*.

[35] McCaffery I., Tudor Y., Deng H. (2013). Putative predictive biomarkers of survival in patients with metastatic pancreatic adenocarcinoma treated with gemcitabine and ganitumab, an IGF1R inhibitor. *Clinical Cancer Research*.

[36] Pan S., Chen R., Crispin D. A. (2011). Protein alterations associated with pancreatic cancer and chronic pancreatitis found in human plasma using global quantitative proteomics profiling. *Journal of Proteome Research*.

[37] Wlodarczyk B., Borkowska A., Wlodarczyk P., Malecka-Panas E., Gasiorowska A. (2020). Serum levels of insulin-like growth factor 1 and insulin-like growth factor-binding protein 2 as a novel biomarker in the detection of pancreatic adenocarcinoma. *Journal of Clinical Gastroenterology*.

[38] Grimberg A., Coleman C. M., Shi Z., Burns T. F., Mac Lachlan T. K., Wang W. (2006). Insulin-like growth factor binding protein-2 is a novel mediator of p53 inhibition of insulin-like growth factor signaling. *Cancer Biology & Therapy*.

[39] Gao S., Sun Y., Zhang X. (2016). IGFBP2 activates the NF-*κ*B pathway to drive epithelial-mesenchymal transition and invasive character in pancreatic ductal adenocarcinoma. *Cancer Research*.

[40] Li X., Ma Q., Xu Q. (2012). SDF-1/CXCR4 signaling induces pancreatic cancer cell invasion and epithelial-mesenchymal transition _in vitro_ through non-canonical activation of hedgehog pathway. *Cancer Letters*.

[41] Das S. K., Bhutia S. K., Azab B. (2013). MDA-9/syntenin and IGFBP-2 promote angiogenesis in human melanoma. *Cancer Research*.

[42] Dahlem C., Barghash A., Puchas P., Haybaeck J., Kessler S. M. (2019). The insulin-like growth factor 2 mRNA binding protein IMP2/IGF2BP2 is overexpressed and correlates with poor survival in pancreatic cancer. *International Journal of Molecular Sciences*.

[43] Ho J. J., Siddiki B., Kim Y. S. (1995). Association of Sialyl-Lewis(a) and Sialyl-Lewis(x) with MUC-1 apomucin in a pancreatic cancer cell line. *Cancer Research*.

[44] Ballehaninna U. K., Chamberlain R. S. (2012). The clinical utility of serum ca 19-9 in the diagnosis, prognosis and management of pancreatic adenocarcinoma: an evidence based appraisal. *J Gastrointest Oncol*.

[45] Johansson K., Kaprio T., Nieminen H. (2022). A retrospective study of intraductal papillary neoplasia of the pancreas (IPMN) under surveillance. *Scandinavian Journal of Surgery*.

[46] Kim S. S., Lee S., Seung Lee H., Bang S., Han K., Park M. S. (2022). Retrospective evaluation of treatment response in patients with nonmetastatic pancreatic cancer using CT and CA 19-9. *Radiology*.

